# UV induced resistive switching in hybrid polymer metal oxide memristors

**DOI:** 10.1038/s41598-020-78102-x

**Published:** 2020-12-03

**Authors:** Spyros Stathopoulos, Ioulia Tzouvadaki, Themis Prodromakis

**Affiliations:** Centre for Electronics Frontiers, Zepler Institute for Photonics and Nanoelectronics, University of Southampton, Southampton, SO17 1BJ UK

**Keywords:** Engineering, Nanoscience and technology

## Abstract

There is an increasing interest for alternative ways to program memristive devices to arbitrary resistive levels. Among them, light-controlled programming approach, where optical input is used to improve or to promote the resistive switching, has drawn particular attention. Here, we present a straight-forward method to induce resistive switching to a memristive device, introducing a new version of a metal-oxide memristive architecture coupled with a UV-sensitive hybrid top electrode obtained through direct surface treatment with PEDOT:PSS of an established resistive random access memory platform. UV-illumination ultimately results to resistive switching, without involving any additional stimulation, and a relation between the switching magnitude and the applied wavelength is depicted. Overall, the system and method presented showcase a promising proof-of-concept for granting an exclusively light-triggered resistive switching to memristive devices irrespectively of the structure and materials comprising their main core, and, in perspective can be considered for functional integrations optical-induced sensing.

## Introduction

Light-induced programming or combinational schemes involving both light and electrical stimulation have been studied for different applications from switches^1,2^ to neuromorphics^3,4^. Moreover, resistive switching has been demonstrated in various configurations; ethanol-adsorbed ZnO thin film upon visible light activation^5^, ultra-thin hafnium-oxide^6^, multiferroic thin film memristors^7^, ITO/oxide devices^8^, as well as in RRAM devices through combination of light and electrical stimuli using a thin SiO_x_ layer sandwiched between a transparent top electrode and a p-type Si substrate^9^. Furthermore, other configurations involving various nanostructures like semiconductor quantum dots, nano-rods and Metal–Insulator–Semiconductor structures have been studied for light-controlled resistive switching^10–14^ or enhancement of the resistance of a conducting filament in the dielectric^15^.

Meanwhile, conductive and semiconducting polymers have shown a dynamic involvement in the area of electronics holding active roles as organic semiconductors, electrodes or intermediate layers in Organic Field Effect Transistors (OFETs)^16^, Organic Light Emitting Diodes (OLEDs)^17^ and Organic Photovoltaics (OPVs)^18^, or assisting the functions of other configurations, for example when used for electromagnetic shielding^19^. Among those, poly (3,4ethylenedioxythiophene):poly(styrenesulfonate), an intrinsically electrically conducting polymer also known as PEDOT:PSS, is a widely used material in flexible and printed electronic devices^20^.

Metal–insulator memristors^21^ (pristine MIM) are subjected to surface treatment of the top-electrode with PEDOT:PSS and resulted in a PEDOT:PSS/MIM hybrid system. In contrast to a bottom-up approach, where the device is designed for light stimulation ab initio (as for example involving silicon^9^) the functionalization method presented here provides the capability to re-purpose existing MIM devices and enhance them with light-assisted programming, while retaining their CMOS compatibility.

**Figure 1:**
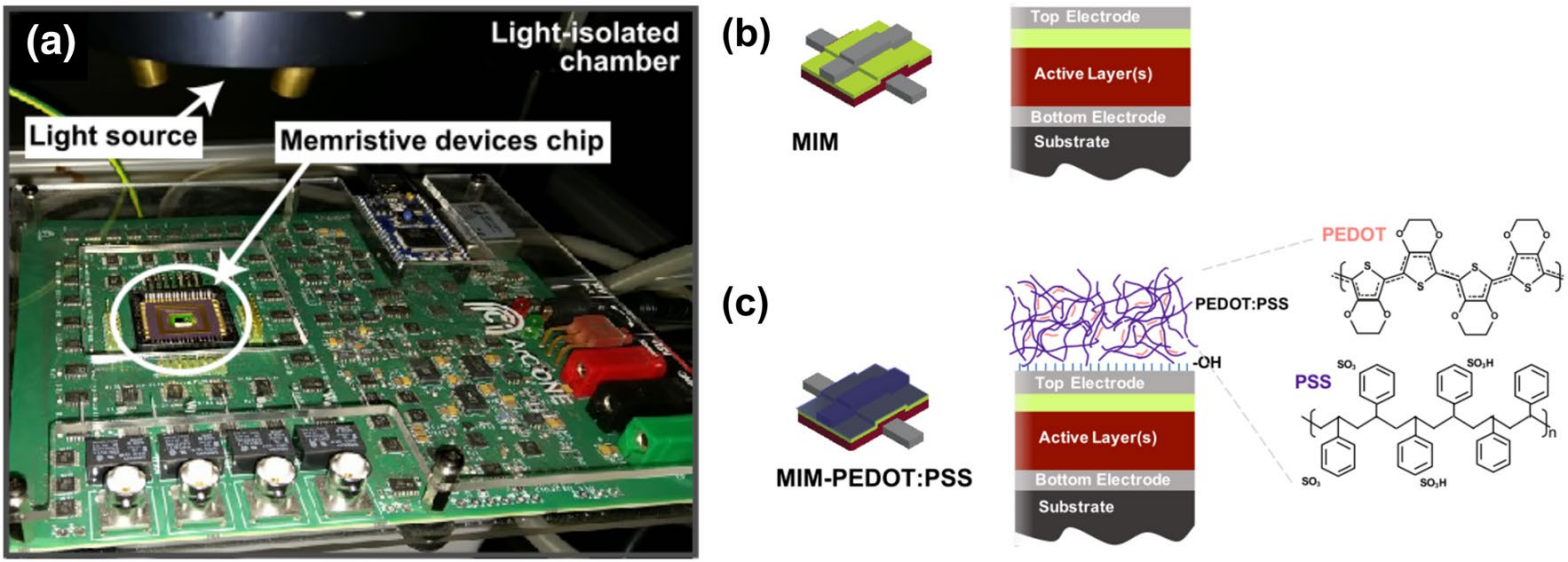
Illustration of the experimental setup. (**a**) The device plugged into the characterization platform inside the light-isolated chamber (green light is used for guidance only and is disabled during illumination). (**b**) Schematic cross-section of the pristine MIM device comprising a Pt/TiO_2_/Al_2_O_3_/Pt structure. **c** Schematic cross-section of the PEDOT:PSS/MIM hybrid structure.

While the MIM memristors show no response upon illumination with respect to their resistive state level, steep steps of increasing resistive state values are recorded for the case of PEDOT:PSS/MIM hybrid device upon exposure to optical stimulation with UV light of a 300 nm for 600 s. This can be attributed to the photon absorption taking place in the PEDOT:PSS, which affects the charge distribution in the organic PEDOT:PSS layer, and subsequently through interfacial carrier transport layers effects^22,23^ ultimately amends the electrical characteristics of the MIM device. Although the stimulation is not done directly electrically it does conform to the generalised concept of the memristor as described by Chua^24^ by means of modulating its internal state, (i.e. its resistance). Figure 1 illustrates the experimental setup and the functionalization principle for the devices used throughout this paper.

**Figure 2:**
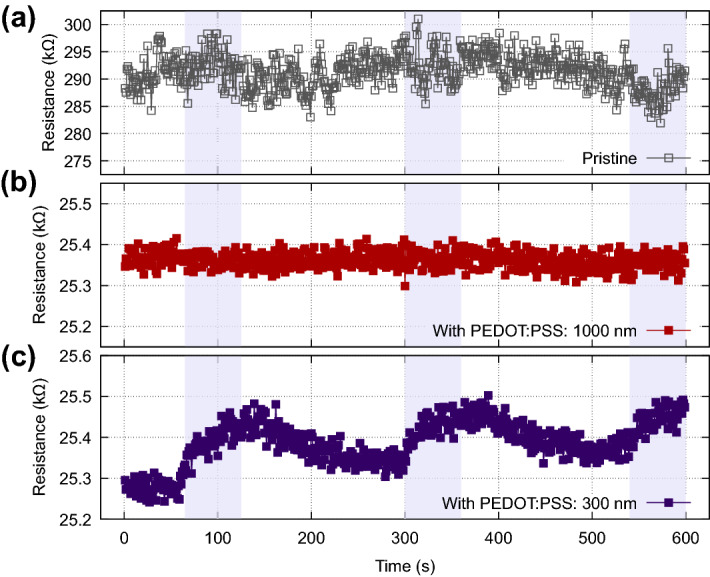
Different illumination regimes compared to the pristine MIM device**. **(**a**) Pristine MIM device exposed to 300 nm. (**b**) PEDOT:PSS/MIM hybrid device exposed to 1000 nm and (**c**) PEDOT:PSS/MIM hybrid device exposed to 300 nm. Pristine MIM devices do not indicate significant changes in their resistive state upon UV-illumination. No change in the resistive state was recorded for the PEDOT:PSS/MIM hybrid device when exposed to the infrared wavelength (1000 nm). On the contrary, when the PEDOT:PSS/MIM hybrid is exposed to UV wavelengths there is an apparent change in the resistance. Highlighted regions indicate active illumination.

In order to further investigate the effect of the optical stimulation to resistance switching, real-time measurements are performed, for the hybrid PEDOT:PSS/MIM system combined with subsequent exposure to stimulation with light sources of different wavelengths. As control experiment the same illumination protocol was followed in pristine MIM devices. As can be seen in Fig. 2, pristine devices elicit no response upon illumination. This is also the case for the hybrid PEDOT:PSS/MIM device when exposed in infrared. On the other hand, a prominent response is recorded when the hybrid PEDOT:PSS/MIM device is exposed to UV radiation.

**Figure 3:**
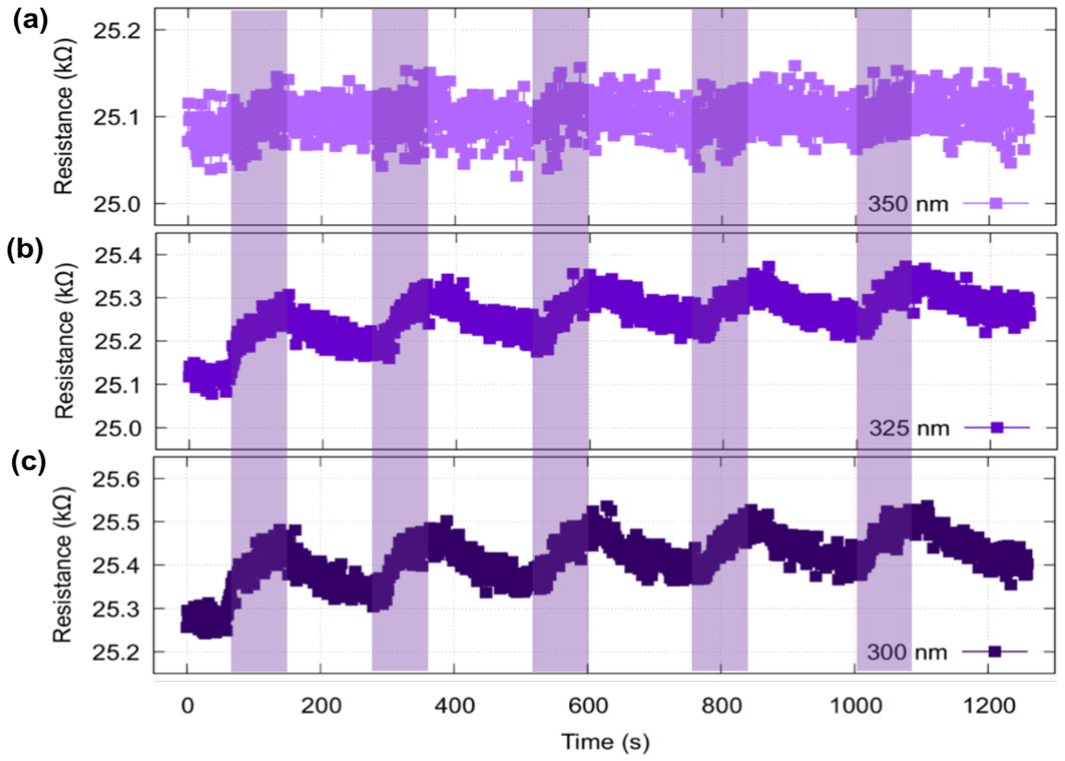
Resistive state response and wavelength dependence upon light exposure. (**a**) 350 nm, (**b**) 325 nm and (**c**) 300 nm for the PEDOT:PSS/MIM hybrid device. For increasingly higher energy photons the change in resistance is more pronounced. In all cases we used alternating light exposure (60 s)—dark (150 s) for a total experiment time of 20 min. Highlighted regions indicate active illumination.

Since a significant response was recorded in the UV range, we proceeded to scan across different UV wavelengths, considering that the intensity value is constant in the range of wavelengths considered. First, the lowest energy UV light (350 nm) is investigated. A very small almost insignificant difference is recorded at the resistive state level (Fig. 3). However, when decreasing the wavelength (325 nm) a resistive state change is introduced and becomes even more pronounced at the lowest wavelength (300 nm) demonstrating a clear wavelength specificity.

**Figure 4:**
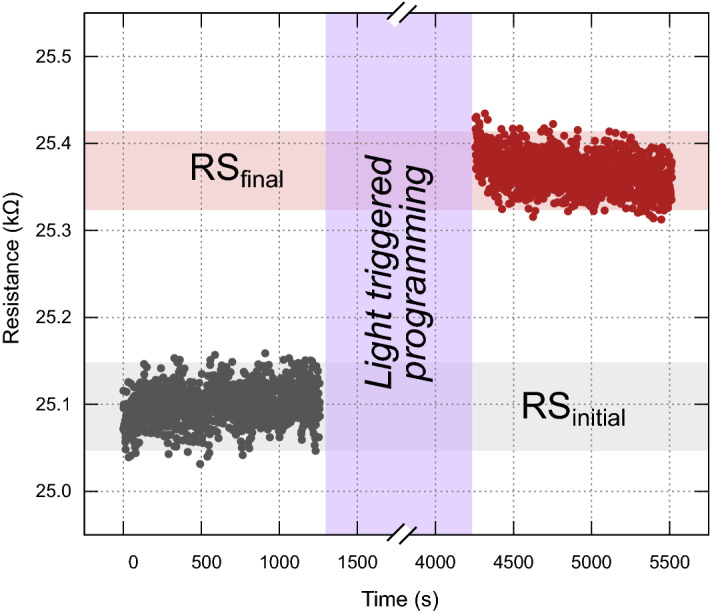
Resistive switching results. Transition from an initial resistive state regime to an approximately 300 Ω higher resistive state level achieved using only repetitive light-triggered programming cycles.

The light-induced switching approach is evaluated for its capacity to program the PEDOT:PSS/MIM hybrid memristor and to achieve a resistive multi-state spectrum within a resistive change range, by repetitive light-triggered programming cycles of 210 s each. Each cycle consists of 60 s of illumination-stimulation (light-soaking) and is then followed by 150 s of darkness-relaxation. The stimulation and relaxation timeframes are chosen as to be the minimum duration required for ensuring a new distinct resistive state. At the beginning, a gentler programming mode is considered through the choice of a 325 nm wavelength light and then a slightly more intense mode of 300 nm wavelength is applied for reaching the desired resistive state depicting a difference between initial and final resistive state (ΔR) of approximately 300 Ω (Fig. 4). The resistive switching procedure stops as soon as the UV-light source is switched-off and the levels of the state variable remain constant for the following 20 min (time chosen to be equal with the total stimulation time while UV-light source was activated) of reading pulses only and keeping the UV-light source deactivated. The light/PEDOT:PSS interaction has been directly translated into a recordable change in the resistive state of the device regardless of the actual underlying physicochemical mechanism.

In voltage-pulse programming, a specific resistive state can be achieved by modulating the voltage pulse characteristics such as the number of pulses the pulse width and amplitude. Similarly, in the illumination-pulse approach, as hereby demonstrated, different modes of programming and a desired state can be achieved by modulating the wavelength (and consequently the energy of the photons), the duration of the illumination exposure and the number of light-exposure events. Therefore, tuning these parameters may allow tailoring the resistive switching process, enabling discrimination of resistive states in chosen resistive levels. Furthermore, conventional approaches of sequential pulsing of the device apart from the fact that they continuously necessitate the application of a voltage stimulus, they can often be aggressive particularly for devices with initially low resistive states and can also potentially mask valuable resistive levels. On the contrary a light-triggering switching approach may bring significant benefits for cases requiring a gentler programming procedure, for example when very low initial states are involved or for protecting a device from abrupt resistance changes and risk of degeneration of the memristive character or conversion of the device to an Ohmic element during the programming. Additionally, a light stimulus may help for lowering the cross-talk in more complex integrations, an important feature for building efficient brain like computing networks, and for elimination of electrical Joule heating. Our approach introduces light-sensitivity to a well established device structure with minimal additional processing, when compared to existing works (Table 1), and it is demonstrated to record a resistive response even at low incident power densities. Despite the fact the we demonstrated only a proof-of-concept of the approach, this concept can be expanded to program dense device arrays by exposing all the devices simultaneously to the chemical surface treatment and subsequent UV-light exposure. This can open the way for many implementations such as functional integration including optical signal sensing, imagers, or as an indicator for potential UV-corrosions in various vulnerable to radiation products. It is important to mention that in this work the device still requires the use of a negative polarity pulse to reset it back to its initial state. For a vertically stacked bipolar device as the one used in the paper there is no direct way to directly functionalise the bottom electrode so as to allow us tuneable control of both electrodes. However this can be overcome by utilising unipolar devices where the device would reset back to its original state after a resistive threshold has been crossed for a fully optical programming process or by using planar devices where both electrodes are directly exposed.

**Table 1:** Light-tunable resistive switching overview.

Structure	Trigger	Fabrication requirements
Cu/MoS_2_ NRs/Pt^14^	White light (50 W/m^2^) and electrical	Nanorods
ITO/SiO_x_/p-Si^9^	VIS/IR light (410–1100 nm; 0.8 μW) and electrical	MOS (p-Si)
ITO/ZnO/p-Si^25^	VIS light (532 nm; 300 mW/cm^2^) and electrical	MOS (p-Si)
Ag/BiFeO_3_/ZnO/FTO^26^	LED (35 W) and electrical	BiFeO_3_–ZnO heterojunction; multiferroic material
Pt/Al_2_O_3_/SiO_2_/Si^13^	UV and IR LED (2.5 mW/cm^2^) and electrical	MOS (p-Si)
QD/GaAs/AlGaAs^10^	CW illumination (2 eV; 730 nW; 44 μW), IR (1.32 eV; 2.2 mW–3.6 mW) and electrical	InAs QDs and predefined hole structures
Al/PMMA/ZPNPs/PMMA/ITO/QZ^3^	VIS and UV light (0.05 mW/cm^2^) and electrical	ZnO-monolayer phosphorene NPs
Al/BMThCE/ITO/QZ^27^	UV/VIS light (5.86 mW/cm^2^) and electrical	Photochromophore (BMThCE)
Au/HFO/SiO_2_Si^6^	VIS light (45 mW) and bias electric field	High-k HFO layer and semi-transparent Au top electrode
ITO/HfO_2_/ITO^8^	Blue (65 mW/cm^2^) and red (104 mW/cm^2^) mediated negative conductivity; SET/RESET cycles by DC voltage/light cycles	Transparent oxides
Pt/BaTiO_3_/NiFe_2_O_4_/BaTiO_3_/Au^7^	UV (365 nm at 11.5 mW/cm^2^, 302 nm at 3.78 mW/cm^2^) and electrical	Multiferroic material
Au/ZnONRs/FTO/QZ^11^	UV/VIS/IR (200–2500 nm); 300 W xenon light source	Nanorods
Ag/BiFeO_3_/γ-Fe_2_O_3_/FTO^12^	White light (20 mW/cm^2^) and electrical and magnetic field UV (300–350 nm) stimulus (7 mW/cm^2^)	Multiferroic material
Pt/AlO_3_/SiO_2_/Au (this work)	UV (300–350 nm; 7 mW/cm^2^) and electric reset	Top electrode functionalization

While other methods generally report better ON/OFF ratios, they also require additional fabrication complexity such as introduction of nanoscale elements or semiconductive materials. Moreover, as usually reported, the light switching is further supported by electrical stimuli or/and a high-power light source. On the contrary, the proposed method offers a versatile method for inducing resistive switching requiring a straightforward post-fabrication functionalization of the top electrode with standard drop casting and by only applying UV-light stimulation of low power source for inducing resistive switching without any additional voltage stimuli. Overall, the proposed method results in a soft programming procedure, also offering significant system scalability along with the possibility for multi-panel photo-sensitive arrays through the selective functionalization of distinct devices.

In conclusion, we show a new and straight-forward, UV-light-exclusively method for programming memristive devices. An elaborated version of a metal-oxide memristive architecture is introduced, comprising a hybrid top electrode obtained through treatment with PEDOT:PSS. Light-soaking, hereby considered as an *illumination-pulse* triggering condition, with wavelengths belonging to UV spectrum, ultimately resulted to resistive switching, also demonstrating a direct relation between the switching magnitude and the applied wavelength. This approach does not involve any additional voltage stimulation for setting the devices at a specific resistive value, providing an alternative and less aggressive programming procedure. Finally, this study further highlights the importance of exploring new strategies, chemistries and materials for tailoring the resistive switching performance.

## Methods

### MIM memristors pristine system

Two-terminal MIM devices are realized on a 6-in. oxidized silicon wafer (200 nm of dry thermal SiO_2_). First, the fabrication of 20 μm wide bottom electrodes is performed using a photolithography process followed by electron beam evaporation of a 5 nm titanium (Ti) adhesion layer and 10 nm of platinum (Pt). What follows is a lift-off process in N-Methyl-2-pyrrolidone (NMP) and then, 25 nm of titanium dioxide (TiO_2_), as the solid electrolyte, are deposited using reactive magnetron sputtering from Ti target in an 8 sccm oxygen (O_2_) environment. A 4 nm aluminium oxide (Al_2_O_3_) layer, acting as the interface barrier layer for providing a final bilayer configuration, is also deposited using the same process without breaking the vacuum. The active layer is defined with negative tone photolithography. The top 20 μm wide Pt electrodes (10 nm) are also formed with electron beam evaporation and lift-off. Active area of the final device is 20 × 20 μm^2^. Following the fabrication process, the wafer is diced into chips of 3 × 3 mm^2^. Each single chip, consisting of multiple MIM devices, is wire-bonded to a commercially provided ceramic quad flat J-shaped (CQFJ) chip-holder with connections suitable for the memristor characterization platform that is used. The MIM devices are initially electroformed for demonstrating hysteretic characteristics and bipolar behaviour as per, and brought to a resistive level between 25 and 300 kΩ. Electrical characteristics for this class of devices and at similar resistive ranges has been presented elsewhere^21,28,29^. For this process the device is subjected to consecutive 10–100 μs pulses of negative polarity ranging from − 3 to − 8 V with a 0.25 V voltage step. Interval between pulses (interpulse time) has been kept constant at 10 ms. Devices have not been subjected to any further forming steps during the course of the experiment.

### PEDOT:PSS/MIM hybrid system

For the surface modification, the pristine MIM devices are treated with oxygen plasma for 15 min (30 sccm, 99 mTorr) in order to generate hydroxyl-terminating groups (OH) on the surface. The purpose of the plasma treatment is twofold: it clears any remaining organic residue as well aids in better adhesion of the polymer to the electrode surface. The device is then functionalized by exposure of the OH-activated surface to 1 μL of PEDOT:PSS (655201-5G, Sigma-Aldrich) using standard drop-casting.

### Experimental set-up

The complete system is placed inside an isolated dark chamber (as illustrated in Fig. 1) in such way that the chip is directly exposed to a Bentham ILD-Xe-QH Xenon-QTH light source. The light source is coupled with a TMC300 monochromator so that the setup is able to provide single wavelength light in the range from 250 to 2500 nm. For the wavelengths used in this work output power density is 6–8 mW/cm^2^ in the UV range and 400 mW/cm^2^ for the infrared illumination. The chip is always plugged into the custom-made hardware^30^ supported by custom-made software^21^ (Fig. 1a) which for the purposes of the present study is used only for recording and providing the electrical readout of the resistance over time (retention). The devices are subjected to non-switching pulses that allow the readout without affecting the state (fixed reading voltage pulse of 200 mV). Sampling is performed every 1 s in a real-time way, while alternating an exposure-non-exposure cycle with a light of a chosen wavelength. The monitoring procedure always starts with the light source in an inoperative state for 60 s in order to record the initial resistive state of the system and gauge its stability. Then the light source is activated and the system is exposed to it for 60 s. Four different wavelengths are studied in total, three within the ultraviolet spectrum (i.e. 300 nm, 325 nm and 350 nm) and one in near infrared region (i.e. 1000 nm). Then the light source is deactivated for the next 150 s and the resistive state is monitored in the dark until the light is anew activated for 60 s. The before-mentioned cycle is repeated multiple times for a total time of 20 min for each wavelength.
