# Author Correction: UV induced resistive switching in hybrid polymer metal oxide memristors

**DOI:** 10.1038/s41598-021-86897-6

**Published:** 2021-03-26

**Authors:** Spyros Stathopoulos, Ioulia Tzouvadaki, Themis Prodromakis

**Affiliations:** Centre for Electronics Frontiers, Zepler Institute for Photonics and Nanoelectronics, University of Southampton, Southampton, SO17 1BJ UK

Correction to: *Scientific Reports* 10.1038/s41598-020-78102-x, published online 03 December 2020

This Article contains an error. A Data Availability section was originally not included—it should appear as below:

## Data Availability

The data that support the findings of this study are available from the University of Southampton institutional repository at: 10.5258/SOTON/D1637

